# Cognitive outcomes after tadalafil treatment in patients with cerebral small vessel disease: ETLAS-2 sub-study

**DOI:** 10.1016/j.cccb.2025.100520

**Published:** 2025-11-11

**Authors:** Joakim Ölmestig, Kristian N. Mortensen, Marie B. Thomas, Birgitte Fagerlund, Trevor W. Robbins, Nadia Naveed, Mette M. Nordling, Hanne Christensen, Helle K. Iversen, Mai B. Poulsen, Hartwig R. Siebner, Christina Kruuse

**Affiliations:** a Neurovascular Research Unit, Department of Neurology, Copenhagen University Hospital – Herlev and Gentofte, Borgmester Ib Juuls Vej 1, 2730 Herlev, Denmark; b Department of Clinical Medicine, University of Copenhagen, Blegdamsvej 3B, 2200 Copenhagen, Denmark; c Danish Research Centre for Magnetic Resonance, Department of Radiology and Nuclear Medicine, Copenhagen University Hospital – Amager and Hvidovre, Nordre Hospitalsvej 13, 2650 Hvidovre, Denmark; d Child and Adolescent Mental Health Center, Copenhagen University Hospital, Mental Health Services CPH, Kildegaardsvej 28, 2900 Hellerup, Denmark; e Department of Psychology, University of Copenhagen, Øster Farigmagsgade 2A, 1353 Copenhagen, Denmark; f Behavioural and Clinical Neuroscience Institute, Department of Psychology, University of Cambridge, Downing Street, Cambridge CB2 3 EB, United Kingdom; g Department of Radiology, Copenhagen University Hospital – Herlev and Gentofte, Borgmester Ib Juuls Vej 1, 2730 Herlev, Denmark; h Department of Neurology, Copenhagen University Hospital – Bispebjerg and Frederiksberg, Bispebjerg Bakke 23, 2400 Copenhagen, Denmark; i Department of Neurology, Copenhagen University Hospital – Rigshospitalet, Valdemar Hansens Vej 13, 2600 Glostrup, Denmark; j Department of Neurology, Copenhagen University Hospital – North Zealand, Dyrehavevej 29, 3400 Hillerød, Denmark; k Department of Brain and Spinal Cord Injury, Neuroscience Centre, Copenhagen University Hospital – Rigshospitalet, Valdemar Hansens Vej 23, 2600 Glostrup, Denmark

**Keywords:** CANTAB, MoCA, SDMT, Trail-making test A and B, Digit span, Verbal fluency

## Abstract

**Background:**

Cerebral small vessel disease (CSVD) is the dominant cause of vascular cognitive impairment and dementia. We aimed to test whether continuous daily treatment with the vasoactive phosphodiesterase 5 inhibitor tadalafil improved cognitive performance in patients with CSVD.

**Methods:**

This is a pre-registered sub-study on cognitive outcomes from the ETLAS-2 trial, a randomized, placebo-controlled, double-blind, parallel study investigating 3 months of daily tadalafil (20 mg) treatment against placebo in patients with CSVD and previous stroke or transient ischemic attack. Outcomes were assessed twice, at baseline and after 3 months of treatment. The primary outcomes were changes in the cognitive domains of processing speed, attention, memory, and executive functions assessed by the Symbol Digit Modalities test, digit span forward, and tests from the computer-based Cambridge Neuropsychological Test Automated Battery (CANTAB).

**Results:**

The per-protocol sub-study analysis comprised 60 participants, 28 of whom received tadalafil and 32 received placebo. Baseline cognitive impairments involved reduced processing speed and impaired visual learning and memory. At follow-up, we found a slower mean simple reaction time in the tadalafil group compared to placebo (28.8 ± 13.8 ms, *p* = 0.04). There were no differences between groups in any other primary or secondary outcome.

**Conclusion:**

We found no overall difference in cognitive outcomes between groups over 3-month treatment. In the tadalafil group, a slower reaction time in a single test was observed, which may be a spurious finding. A longer treatment time may be required to identify potential effects on cognitive outcomes, which could be secondary to improved blood flow.

**Clinical trial registration:**

URL: https://www.clinicaltrials.gov, Unique identifier: NCT05173896.

## Highlights

• Three months of tadalafil did not improve cognition.
• Cerebral small vessel disease was linked to reduced processing speed.
• Longer PDE-5 inhibitor treatment duration is warranted in future trials.

## 1 Introduction

Cognitive impairment is commonly associated with longstanding cerebral small vessel disease (CSVD), which is widely recognized as a primary contributor to vascular cognitive impairment (VCI) and vascular dementia (VD). Dementia disorders represent significant global health challenges due to their high prevalence and impact on individuals and healthcare systems [1]. An effective treatment for cognitive impairment associated with CSVD has not yet been identified [2]. Recently, the LACI-2 trial showed reduced cognitive impairment after nitric oxide (NO) donor treatment for 12 months in participants with CSVD [3]. In this trial, we targeted the same vasodilatory cyclic guanosine monophosphate (cGMP) pathway downstream by use of tadalafil, an inhibitor of the cGMP-degrading enzyme phosphodiesterase 5 (PDE5).

Cognitive functions are commonly categorized into domains, including memory, executive functions, language, processing speed, and attention [4]. CSVD is associated with cognitive impairments in multiple domains [5], but mainly manifests as impaired executive functioning, processing speed, and attention [4]. CSVD pathophysiology is suggested to be initiated by changes in the vascular endothelium with impaired NO—cGMP signaling, leading to reduced vascular function [6]. Thus, targeting the NO—cGMP pathway pharmacologically may be a viable method for enhancing vascular function, which could yield positive benefits on cognition.

This paper presents the cognitive outcomes from the ETLAS-2 sub-study, which investigated the effects of daily tadalafil versus placebo treatment for 3 months in individuals with CSVD and a history of stroke or transient ischemic attack (TIA). The results are considered exploratory, with the primary aim of assessing whether short-term tadalafil treatment affects cognitive function in this population, specifically in the cognitive domains of processing speed and executive function.

## 2 Materials and methods

### 2.1 Study design

In the ETLAS-2 Trial, we investigated the effect of a 3-month daily oral tadalafil intake (20 mg) versus placebo on clinical, cognitive, and neurovascular outcomes, as reported in the published trial paper [7]. The trial was conducted as a randomized, placebo-controlled, double-blind, parallel-arm study. The trial was conducted in compliance with relevant laws and institutional guidelines and was approved by the Ethics Committee in the Capital Region of Denmark (H–20031301), the Danish Medicines Agency (EudraCT: 2020–002329–27), and the Capital Region at the Knowledge Center for Data Reviews (P-2020–1193). The trial was monitored by the Good Clinical Practice (GCP) unit in Denmark and registered at clinicaltrials.gov (NCT05173896) prior to trial initiation. All participants who were enrolled in the ETLAS-2 Trial were automatically enrolled in the cognitive sub-study, and all participants provided written informed consent prior to participation. The trial was conducted in a collaborative network in the Capital Region of Denmark. The main site was the Dept. Neurology, Copenhagen University Hospital – Herlev and Gentofte (HGH), where patient recruitment, clinical visits, and cognitive testing were done. Study MRIs were conducted at the Danish Research Centre for Magnetic Resonance (DRCMR), Copenhagen University Hospital – Amager and Hvidovre (AHH). Participants were also recruited among patients previously admitted with stroke at the Dept. Neurology, Copenhagen University Hospital – North Zealand (NZH), Dept. Neurology, Copenhagen University Hospital – Bispebjerg and Frederiksberg (BFH), and Dept. Neurology, Copenhagen University Hospital – Rigshospitalet (RH). Once the planned sub-studies have been published, deidentified participant data will be made available upon reasonable request. Requests should be emailed to the corresponding author.

### 2.2 Procedures

We screened for eligible participants from patients admitted between 6 months and 5 years ago at the Dept. Neurology HGH, NZH, BFH, and RH, as per published inclusion and exclusion criteria [7]. Since tadalafil was not approved for use until 6 months after a stroke, we included stroke/TIA patients in the chronic phase (>6 months ago). Interested participants were invited to an information and inclusion visit, and the included participants underwent a baseline visit directly afterwards, during which cognitive testing was performed. The baseline visit was followed by the first MRI, after which participants began the study medication, administered orally as either tadalafil (20 mg) or placebo. After 3 months, a second MRI was performed, followed by a visit on the final day of study medication, during which cognitive testing was repeated. Randomization was done by the Capital Region Pharmacy [7].

Cognitive testing took approximately 1.5 h and was divided into two sessions with a planned break in between. The participants were instructed to inform the investigator if they needed additional breaks, which was endorsed if required. We conducted paper-pencil cognitive tests in the first session and computer-based tests in the second session (Table 1). All tests were done in the same order and in the same examination room on both trial days. We encouraged the participants to complete all tests. However, if they objected and did not want to continue a test, the test was aborted. The computer-based tests were from the Cambridge Neuropsychological Test Automated Battery (CANTAB) (Cambridge Cognition, UK), using the PC-based CANTAB Eclipse version, which employed a touchscreen. All the cognitive examiners were clinical study personnel trained in the specific tests by an experienced neuropsychologist. The Montreal Cognitive Assessment (MoCA) was selected as a basic cognitive screening tool for assessing various cognitive domains. The other paper-pencil and CANTAB tests were specifically selected (by co-authors BF and TR) to investigate cognitive domains that are known to often be impaired in CSVD, including processing speed, executive function, and attention [4].

**Table 1:** List of cognitive tests performed in the ETLAS-2 Trial in sequential order.

	Test	Estimated duration	Cognitive domain
Paper-pencil tests	Montreal Cognitive Assessment (MoCA)	10 min	Memory, executive functioning, attention, language, visuospatial, and orientation [8]
	Symbol Digit Modalities Test (SDMT)	2 min	Processing speed [9]
	Trail-making test A & B	5 min	Executive function, processing speed [10]
	Verbal fluency (animal, F-A-S words)	5 min	Language, verbal fluency [11]
	WAIS-IV: digit span forwards, backwards, ordering, and letter-number sequence	10 min	Verbal working memory, attention [12]
	Danish Adult Reading Test	3 min	Pre-morbid intelligence [13]
Cambridge Neuropsychological Test Automated Battery (CANTAB)	Motor screening task (MOT)	2 min	Sensorimotor function [14]
	Spatial working memory (SWM) 3,4,6,8 boxes.	6 min	Spatial working memory, executive function [15,16]
	Paired associates learning (PAL) 1,2,3,6,8 patterns.	8 min	Visual memory and learning [15,17]
	Reaction time (RTI)	3 min	Attention, motor and mental speed, reaction time [15,18]
	One touch stockings of Cambridge (OTS)	10 min	Executive function, planning, working memory [19]
	Rapid visual information processing (RVP)	7 min	Attention, executive function [15,20]

### 2.3 Participants

We included patients previously admitted with stroke or TIA aged ≥50 years with concomitant signs of CSVD on brain imaging. These signs were defined as an acute or old small vessel occlusion infarct or moderate to severe white matter hyperintensity on a combined deep and periventricular Fazekas scale (≥ Fazekas 2), according to the published trial paper [7].

### 2.4 Outcomes

Primary outcomes: Differences in follow-up results between groups adjusted for the baseline values for the following tests and specific outcomes: MoCA, Symbol Digit Modalities test (SDMT), Digit span forwards, Spatial working memory (SWM, total errors), Paired associates learning (PAL, total errors, adjusted for stages not attempted), Reaction time (RTI, mean simple reaction time), One touch stockings of Cambridge (OTS, mean choices to correct), and Rapid visual information processing (RVP, signal detection measure A’). The results of the MoCA test are also presented in the ETLAS-2 main study [21].

Secondary outcomes: Differences in follow-up results between groups adjusted for the baseline values for the following tests: Trail-making test A, Trail-making test B, Trail-making test (total), Trail-making B/A ratio, Verbal fluency (F), Verbal fluency (A), Verbal Fluency (S), Verbal fluency (animals), Verbal fluency (total), Digit span backwards, Digit span ordering, Digit span letter-number sequence, Digit span (total), SWM (between errors), SWM (strategy), PAL (total trials), PAL (first trial memory score), RTI (mean simple movement time), RTI (mean five choice reaction time), RTI (mean five choice movement time), OTS (problems solved on first choice), OTS (mean latency to correct), and RVP (mean latency).

Post-hoc outcomes: The ETLAS-2 study also contained MRI results on white matter hyperintensity (WMH) volume and cerebral blood flow (CBF) in the main study and ETLAS-2 MRI sub-study. We aimed to assess whether interactions between tadalafil treatment and WMH volume changes, as well as between tadalafil and whole-brain CBF changes, affected cognition. This was analyzed post-hoc on all cognitive outcomes.

### 2.5 Statistical analysis and sample size

We assessed all cognitive outcomes twice, at baseline and at the 3-month visit, to investigate cognitive recovery by tadalafil. Only participants who took the study medication from the start until the 3-month visit were included in this per-protocol analysis of cognitive outcomes, as predefined in the trial paper [7]. However, a comparison of non-compliant tadalafil participants to compliant tadalafil participants was done and presented in Section 3.3 and Tables S3 and S4 in the Supplementary material. The analysis of tests was conducted in a prioritized order, starting with the primary cognitive outcomes selected prior to the trial initiation, with a focus on the domains of processing speed, attention, memory, and executive functions related to CSVD. Secondary cognitive outcomes were subsequently analyzed. The selection of cognitive outcomes into primary and secondary was done by the neuropsychologist (co-author BF). All outcomes were analyzed using analysis of covariance (ANCOVA) statistical models to compare the follow-up test scores between treatment groups with the baseline test scores, age, and sex as covariates. Depression scores were without group differences and, therefore, not included in the statistical models as a covariate. However, we included the baseline depression score in a post-hoc test on RTI, mean simple reaction time described in Section 3.1, to investigate if depression was a confounder of the treatment. If a statistically significant result was found for the study treatment in the ANCOVA analysis, we confirmed whether the model met the assumptions for the ANCOVA analysis. If assumptions were violated, the test follow-up and baseline variables were logarithmically transformed and analyzed again with additional model assumption validation. Participants who did not complete a specific test were excluded from that specific analysis. The number of participants in each group is presented in the outcome tables in the results section (Table 3, Table 4). For the post-hoc analysis of the interaction with tadalafil and changes in WMH/CBF, we first calculated delta values (follow-up minus baseline) for WMH volume, and secondly, for whole-brain CBF. These change variables (ΔWMH and ΔCBF), together with age, were mean-centered prior to inclusion in the mixed-effects models. Separate linear mixed-effects models were fit for ΔWMH volume and ΔCBF. Each model included fixed effects of time (baseline vs. follow-up), treatment group, the centered ΔWMH or ΔCBF variable, and all two- and three-way interaction terms between these factors (treatment × ΔWMH/ΔCBF change, and time × treatment × ΔWMH/ΔCBF). Age and sex were included as covariates. Example of mixed effects model with ΔWMH volume: Outcome ∼ time x treatment x ΔWMH + age + sex + (1 | subject_id). Similar models were also performed using ΔCBF instead of ΔWMH volume. We considered a two-sided *p*-value of 0.05 to be statistically significant. Since this is an exploratory sub-study, we did not correct the analysis for multiple testing as prespecified in the trial paper [7], and missing data were not imputed. Sex in this article refers to biological sex at birth. Analyses were performed using RStudio (version 4.3). The sample size calculation indicated that we needed to include a total of 64 participants to achieve the primary outcome (feasibility of treatment) in the ETLAS-2 main study. We aimed to include 100 participants to account for a potential large drop-out.

## 3 Results

**Fig. 1:**
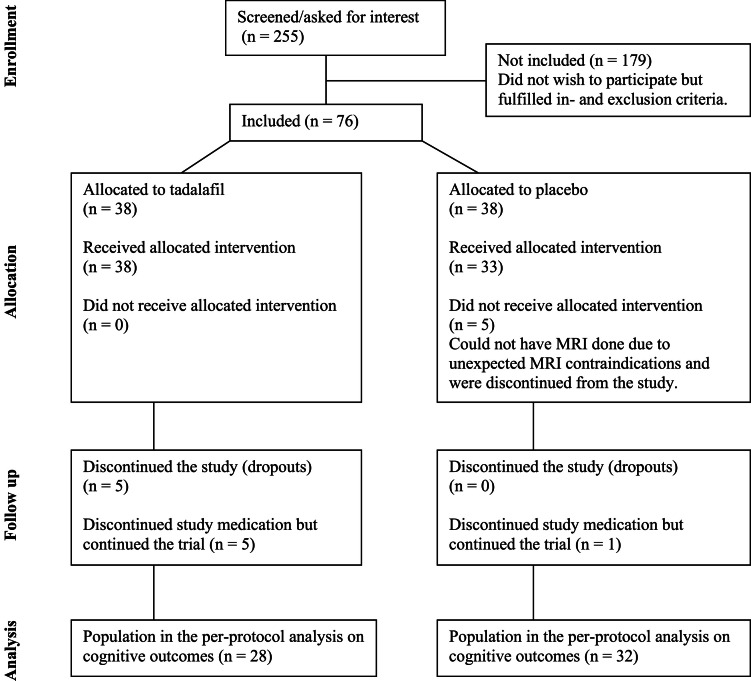
CONSORT flowdiagram.

In this cognitive sub-study analysis based on the per-protocol population, 28 participants received tadalafil and 32 received placebo (Fig. 1). The baseline characteristics of participants are shown in Table 2.

**Table 2:** Characteristics of participants included in the ETLAS-2 cognitive sub-study.

Characteristic	Tadalafil, *N* = 28^1^	Placebo, *N* = 32^1^	*p*-value^2^
Age	68 (60, 72)	66 (62, 75)	0.8
Female	7 (25 %)	7 (22 %)	>0.9
Highest educational level			0.5
Primary school	2 (7.1 %)	0 (0 %)	
Secondary school	1 (3.6 %)	1 (3.1 %)	
Vocational education	5 (18 %)	10 (31 %)	
Short higher education (2–3.5 years)	3 (11 %)	1 (3.1 %)	
Intermediate higher education (3.5–4 years)	9 (32 %)	12 (38 %)	
Long higher education (5–6 years)	8 (29 %)	8 (25 %)	
Hypertension	22 (79 %)	23 (72 %)	0.8
Type 2 diabetes	3 (11 %)	4 (13 %)	>0.9
Hypercholesterolemia	27 (96 %)	32 (100 %)	0.5
Smoking status			0.8
Never	8 (29 %)	10 (31 %)	
Current	5 (18 %)	8 (25 %)	
Previous	15 (54 %)	14 (44 %)	
Ischemic stroke	20 (71 %)	21 (66 %)	0.8
Transient Ischemic Attack	8 (29 %)	11 (34 %)	0.8
Baseline BDI-II score (0–63)	5 (2, 10)	3 (1, 11)	0.7
Baseline FSS score (1–7)	3.44 (2.33, 5.78)	3.78 (2.56, 4.78)	>0.9
Baseline WHO-5 score (0–100)	72 (52, 84)	74 (60, 88)	0.6
Baseline IQCODE score (1–5)	3.06 (3.00, 3.42)	3.06 (3.00, 3.25)	0.5
Baseline DART score (0–50)	22 (13, 28)	20 (14, 28)	>0.9

1 Number or median (Q1, Q3); n (%).

2 Wilcoxon rank sum test; Fisher's exact test. Questionnaire scores are presented with a score range. Abbreviations: BDI-II: Beck Depression Inventory II; DART: Danish Adult Reading Test; FSS: Fatigue Severity Scale; IQCODE: Informant Questionnaire on Cognitive Decline in the Elderly; WHO-5: World-Health Organization 5 – Well-Being Index.

### 3.1 Primary cognitive outcomes

There were no differences between groups in any primary cognitive outcome at follow-up except mean simple reaction time from the RTI CANTAB test (Table 3). Participants in the tadalafil group had 28.8 ± 13.8 milliseconds (*p* = 0.04) longer reaction time compared to placebo. The model did not satisfy the assumption of normality of residuals. A logarithmically transformed RTI score showed a 0.067 ± 0.034 log milliseconds difference (*p* = 0.05) for tadalafil compared to placebo, corresponding to a group difference of 6.97 ± 3.59 % in RTI mean simple reaction time in the tadalafil group compared to the placebo group. This percent difference resembled the increase in the absolute RTI score from the original model. In a post-hoc test on RTI mean simple reaction time, baseline BDI-II score was included as a covariate. In this model, the mean group difference was 33.7 ± 13.2 milliseconds (*p* = 0.01) longer reaction time in the tadalafil group compared to placebo, and BDI-II score was associated with longer reaction time (2.6 ± 1.1 milliseconds per increase in BDI-II score, *p* = 0.03).

**Table 3:** Primary cognitive outcomes.

	Baseline		Follow-up			
Outcome	Tadalafil *N* = 28^1^	Placebo *N* = 32^1^	Tadalafil *N* = 28^1^	Placebo *N* = 32^1^	Mean difference ± SEM^2^	*p*-value
MoCA (n, 0–30)	26 (24, 27)	27 (26, 29)	26 (24, 28)	27 (26, 29)	0.08 ± 0.53	0.88
SDMT (n, 0–110)	35 (31, 44)	38 (35, 43)	36 (29, 45)	40 (35, 44)	–0.39 ± 1.38	0.80
WAIS IV, Digit span forward (n, 0–16)	8 (7, 8)	8 (7, 9)	8 (7, 9)	8 (7, 9)	–0.20 ± 0.38	0.61
SWM, total errors (n)	47 (20, 59)	34 (16, 47)	36 (20, 58)	26 (17, 46)	1.46 ± 3.52	0.68
PAL, total errors adjusted (n)	33 (14, 78)	65 (13, 85)	22 (9, 67) (*n* = 27)	35 (21, 71)	2.69 ± 9.32	0.77
RTI, mean simple reaction time (ms)	376 (337, 400)	363 (310, 399) (*n* = 31)	392 (337, 427) (*n* = 27)	345 (315, 420)	28.79 ± 13.82	0.04
OTS, mean choices to correct (n)	1.40 (1.27, 1.87)	1.40 (1.20, 1.60) (*n* = 31)	1.47 (1.27, 1.80) (*n* = 25)	1.23 (1.13, 1.50)	0.082 ± 0.093	0.38
RVP, A' (n, 0–1)	0.87 (0.85, 0.91) (*n* = 26)	0.89 (0.85, 0.94) (*n* = 29)	0.89 (0.85, 0.91) (*n* = 26)	0.89 (0.82, 0.95) (*n* = 31)	–0.001 ± 0.009	0.90

1 Median (Q1, Q3).

2 The mean difference ± standard error of the mean (SEM) and *p*-values are calculated using the ANCOVA model for that outcome. The mean difference is tadalafil compared to placebo treatment. The number of subjects in each group is stated in the table header. If the number of participants in a test differs from the header number, the actual number of participants is stated within the test row results. Higher MoCA, SDMT, and digit span forward scores are better. Lower SWM total errors, PAL total errors adjusted, RTI mean simple reaction time, and OTS mean choices to correct are better. RVP A’ measures a signal-detection ratio between 0 and 1, a higher score is better. Abbreviations: ms = milliseconds, *n* = the number of scores.

### 3.2 Secondary cognitive outcomes

There were no differences in any of the secondary cognitive outcomes comparing the follow-up test scores between groups adjusted for the baseline test score value, age, and sex (Table 4). The safety of the study medication and side effects are presented and discussed in the ETLAS-2 main study paper [21].

**Table 4:** Secondary cognitive outcomes.

	Baseline		Follow-up			
Outcome	Tadalafil *N* = 28^1^	Placebo *N* = 32^1^	Tadalafil *N* = 28^1^	Placebo *N* = 32^1^	Mean difference ± SEM^2^	*p*-value
Trail-making test A (s)	39 (33, 54)	34 (29, 42)	39 (30, 52)	35 (26, 45)	0.72 ± 2.32	0.76
Trail-making test B (s)	110 (80, 145)	93 (73, 111) (*n* = 31)	98 (73, 130)	92 (64, 120) (*n* = 30)	–0.27 ± 7.35	0.97
Trail-making test total (s, 0–600)	153 (112, 199)	128 (95, 155) (*n* = 31)	134 (101, 183)	120 (90, 160) (*n* = 30)	–3.05 ± 8.35	0.72
Trail-making test B/A ratio	2.76 (2.40, 3.33)	2.76 (2.26, 3.10)	2.48 (2.03, 3.24)	2.62 (2.10, 3.34)	–0.33 ± 0.35	0.35
Verbal fluency, F (n)	11 (9, 12)	12 (9, 15)	11 (8, 14)	12 (9, 15)	0.37 ± 1.01	0.72
Verbal fluency, A (n)	7 (6, 9)	8 (6, 13)	9 (6, 10)	9 (6, 12)	–0.02 ± 0.77	0.98
Verbal fluency, S (n)	11 (9, 13)	13 (9, 15)	10 (9, 14)	13 (8, 14)	0.09 ± 0.95	0.92
Verbal fluency, animals (n)	18 (15, 21)	22 (17, 26)	17 (14, 22)	21 (16, 24)	–1.15 ± 1.00	0.26
Verbal fluency, total (n)	49 (40, 52)	54 (44, 66)	45 (42, 53)	53 (41, 64)	1.49 ± 2.05	0.47
WAIS IV, Digit span backwards (n, 0–16)	6 (6, 8)	7 (6, 7)	6 (6, 8)	6 (5, 8)	0.12 ± 0.43	0.79
WAIS IV, Digit span ordering (n, 0–16)	6 (5, 8)	7 (6, 8)	7 (6, 8)	7 (6, 9)	–0.14 ± 0.42	0.74
WAIS IV, Letter number sequence (n, 0–30)	16 (13, 17)	17 (16, 19)	16 (15, 18) (*n* = 27)	17 (16, 19)	0.74 ± 0.69	0.29
WAIS IV, total score (n, 0–78)	35 (32, 41)	38 (35, 43)	37 (34, 41) (*n* = 27)	38 (35, 43)	1.03 ± 1.23	0.41
SWM, between errors (n)	46 (18, 59)	33 (16, 47)	36 (20, 57)	26 (17, 45)	1.85 ± 3.50	0.60
SWM, strategy (8–56)	37 (33, 41)	36 (28, 38)	35 (32, 40)	34 (26, 37)	0.51 ± 1.63	0.75
PAL, total trials (n)	18 (13, 23)	19 (13, 23)	16 (12, 22) (*n* = 27)	17 (14, 21)	0.27 ± 1.77	0.88
PAL, first trial memory score (n)	17 (13, 20)	16 (13, 19)	16 (14, 20) (*n* = 27)	17 (13, 19)	–0.36 ± 0.92	0.69
RTI, mean simple movement time (ms)	471 (388, 574)	507 (403, 617) (*n* = 31)	483 (410, 549) (*n* = 27)	494 (405, 593)	5.59 ± 25.15	0.82
RTI, mean five choice reaction time (ms)	399 (367, 430)	391 (360, 468) (*n* = 31)	419 (379, 462) (*n* = 27)	423 (363, 452)	–13.47 ± 20.68	0.52
RTI, mean five choice movement time (ms)	487 (444, 569)	462 (392, 552) (*n* = 31)	465 (410, 537) (*n* = 27)	464 (384, 607)	–23.19 ± 27.49	0.40
OTS, problems solved on first choice (n)	8 (7, 12)	11 (8, 12)	10 (8, 12) (*n* = 26)	11 (9, 13)	–0.14 ± 0.63	0.83
OTS, mean latency to correct (ms)	38,722 (25,118, 54,531)	38,559 (28,849, 52,402) (*n* = 31)	33,114 (23,885, 41,602) (*n* = 25)	32,736 (22,854, 51,113)	–751 ± 4129	0.86
RVP, mean latency (ms)	547 (441, 630) (*n* = 26)	522 (417, 589) (*n* = 29)	486 (455, 547) (*n* = 26)	451 (419, 599) (*n* = 31)	–4.35 ± 31.00	0.89

1 Median (Q1, Q3).

2 The mean difference ± SEM and *p*-values are calculated using the ANCOVA model for that specific outcome. The mean difference is tadalafil compared to placebo treatment. If the number of participants in a test differs from the header number, the actual number of participants is stated within the test row results. A trail-making B/A ratio ≥3 reflects executive dysfunction. For verbal fluency, WAIS tests, PAL (first trial memory score), and OTS (problems solved on the first choice), a higher score is better. A lower score is better for the trail-making test, SWM, PAL (total trials), RTI sub-scores, OTS (mean latency to correct), and RVP (latency). ms = milliseconds, *n* = the number of scores, *s* = seconds.

### 3.3 Post-hoc analyses of tadalafil ΔCBF/ΔWMH interactions and drop-out comparisons

A significant tadalafil × ΔWMH interaction indicated that increases in ΔWMH volume were associated with higher (slower) RTI mean simple reaction times in the tadalafil group. When time was included in the model (time × tadalafil × ΔWMH), increases in ΔWMH volume were associated with lower (faster) RTI mean simple reaction times from baseline to follow-up. Similarly, in the tadalafil × ΔCBF interaction, increases in ΔCBF were associated with higher RTI mean simple reaction times, whereas the time × tadalafil × ΔCBF interaction showed that increases in ΔCBF were associated with lower RTI mean simple reaction times from baseline to follow-up. Additional time × tadalafil × ΔCBF interactions indicated that increases in ΔCBF were associated with higher RTI mean five-choice reaction times, higher Trail-making B/A ratios, and lower (worse) OTS problems solved on first choice from baseline to follow-up. In the tadalafil × ΔCBF interaction, increases in ΔCBF were associated with higher RTI mean simple movement time and higher Trail-making test A scores (slower performance). See Table S1 and S2 in Supplementary material for a full list of results from the post-hoc interaction models. Ten participants in the ETLAS-2 trial stopped tadalafil treatment, and five of them completed the follow-up visit without medication. The 10 participants scored similarly on reaction time at baseline but had slightly faster reaction time at follow-up compared to the tadalafil group (Table S3 in Supplementary material). Since there was only follow-up data on five non-compliant participants in the tadalafil group, no statistical comparison to the tadalafil group was done.

## 4 Discussion

In this exploratory ETLAS-2 sub-study, we found no effect of tadalafil on cognitive outcomes in participants with CSVD, except for minor but significant change in RTI (mean simple reaction time), where participants receiving tadalafil had a slower reaction time at follow-up compared to those receiving placebo. This is considered a spurious finding due to multiple comparisons, as no other reaction time test showed any group differences. For completeness, we examined the five non-compliant participants who discontinued tadalafil but completed follow-up. Their reaction times were slightly faster than those of the compliant participants, which could suggest a potential tadalafil-related slowing effect. However, this observation should be interpreted with caution due to the very small sample size. If a true effect were present, the mean group difference of 28.8 ms (6.97 %) would be small and not considered clinically meaningful.

Since depression is known to be associated with psychomotor slowing, we performed a post-hoc test on mean simple reaction time with depression score included as a covariate. Higher baseline BDI-II scores were associated with slower reaction times, but inclusion of BDI-II in the model did not attenuate the treatment effect, suggesting that depressive symptoms did not mediate or confound the observed group difference. The post-hoc mixed models suggest that cognitive outcomes may depend on the interaction between tadalafil and changes in WMH volume and CBF. For RTI (mean simple reaction time), participants with increases in ΔWMH volume or increases in ΔCBF during tadalafil initially had higher reaction times (indicating slower responses) compared with placebo. These effects were attenuated over time, as suggested by the significant three-way interactions. For Trail-making test A and the B/A ratio, larger increases in ΔCBF during tadalafil were associated with higher completion times and ratios (worse score), suggesting an association with task performance. Additionally, for OTS (problems solved on first choice), larger increases in ΔCBF under tadalafil were associated with fewer problems solved over time. Taken together, these findings suggest that cognitive responses to tadalafil may vary depending on changes in CBF and WMH volume. However, these analyses are exploratory, post-hoc, and uncorrected for multiple testing, and should be confirmed in larger studies.

Various CSVD-induced brain injuries (e.g. WMH, lacunae, and enlarged perivascular spaces) have been linked to cognitive impairment in CSVD, probably by disrupting neuronal networks [22]. Evidence suggests that the pathophysiological mechanisms of CSVD originate in the endothelium and are induced by vascular risk factors such as hypertension and diabetes [22]. Endothelial dysfunction is known to lead to blood-brain barrier (BBB) leakage as well as impaired vascular homeostasis and blood flow, through its interaction with vascular smooth muscle cells via the NO—cGMP pathway [2,6]. Impaired BBB and vascular reactivity may be involved in local neuronal injury and pathological changes of CSVD [2]; however, the causality of pathophysiological mechanisms and clinical outcomes is not fully established.

In a systematic review of PDE-5 inhibitors on animal stroke models, multiple studies showed improved memory after PDE-5 inhibitors were administered to rodents, measured with the Morris water maze and aversive radial maze [23]. This improvement was often abolished when simultaneous blockers of upstream or downstream cGMP messengers were co-administered with PDE-5 inhibitors, highlighting the effect of cGMP on cognition [23]. There are only limited studies examining the effect of PDE-5 inhibitors in stroke patients. The PASTIS trial found no significant effect on cognition after a single dose of tadalafil 20 mg versus placebo in patients with CSVD, albeit there was a trend toward improvement in the digit span forward task in favor of tadalafil [24]. Similarly, 3 months treatment with the novel PDE-5 inhibitor PF-03049423 showed no effect on cognition in acute stroke patients [25]. However, promising results on cognition emerged from the LACI-2 trial, which showed reduced cognitive impairment after treatment with isosorbide mononitrate (Imdur) for 1 year in a large population with CSVD [3]. This suggests that prolonged treatment targeting the NO—cGMP pathway may yield beneficial effects on cognitive functioning.

PDE-5 inhibitors have shown positive effects on cognition in individuals with erectile dysfunction (ED). Udenafil (50–100 mg) administered for two months improved Mini-Mental State Examination (MMSE) scores and digit span forward in a pilot study and a subsequent randomized controlled trial [26,27]. Daily tadalafil (5 mg) for 6 months also improved cognitive function in a small study of ED patients with benign prostatic hyperplasia and healthy controls [28]. Additionally, in individuals with ED and mild cognitive impairment, 8 weeks of daily tadalafil (5 mg) improved MoCA scores and CBF assessed by single-photon emission computed tomography imaging [29]. However, most studies reporting cognitive benefits in ED populations lacked placebo controls. In contrast, a placebo-controlled trial in patients with schizophrenia found no cognitive effects of a single sildenafil dose (50–100 mg) [30], and four studies in healthy adults reported no cognitive changes following single doses of sildenafil (100 mg) or vardenafil (10–20 mg) [[31], [32], [33], [34]].

Based on the paper-pencil tests, our population performed well on the MoCA test, with median scores equal to or above the normal score cut-off of 26 points [35]. Additionally, the SDMT scores were comparable to those of a large age-matched healthy cohort [36], as were scores in categorical and phonemic verbal fluency [37]. However, the median group performance in Trail-making test B was slower in our population (median: tadalafil = 110 s; placebo = 93 s) compared to age-matched healthy individuals (median = 76 s), implying reduced processing speed in our cohort [38]. In contrast, our population performed well on digit span forward (median: tadalafil = 8; placebo = 8) and backwards tasks (median: tadalafil = 6; placebo = 7) compared to age-matched controls (mean: 5.56 scores on the forward and 4.18 scores on the backwards tasks) [39] and compared to those with CSVD in the PASTIS trial [24], suggesting intact verbal working memory in our population.

Few studies have applied CANTAB to assess cognition in patients with CSVD, and this trial may thus serve as a valuable reference for future research. The PASTIS trial used the RTI CANTAB test and reported a slower mean simple reaction time (mean: 426 ms) at baseline compared to our population (median: tadalafil = 376 ms; placebo = 363 ms) [24]. Similar to our findings, tadalafil did not improve reaction time in their study [24]. Another study using CANTAB in both CSVD patients and healthy adults found that CSVD was associated with poorer performance on the PAL first-trial memory score (mean score: 7.4) and the A’ measure in the RVP task (mean score: 0.85) compared to healthy controls, suggesting impaired visual learning and memory as well as executive function and attention in CSVD [40]. Notably, the A’ measure in the RVP task performance in that CSVD group [40] was worse than in our population, suggesting that our participants generally had better preserved cognition in the domains of attention and executive functions based on this specific outcome. When compared to normative age-matched (67 years) data from healthy individuals, our population showed prolonged baseline reaction time on the RTI mean simple reaction time test (median: tadalafil = 376 ms; placebo = 363 ms; normative time = 280 ms) and RTI mean five choice reaction time test (median: tadalafil = 399 ms; placebo = 391 ms; normative time = 320 ms) [41]. Further, our population showed poorer scores on PAL mean total errors adjusted (median: tadalafil = 33; placebo = 65; normative score = 23), total trials (median: tadalafil = 18; placebo = 19; normative score = 15), but not PAL first trial memory score (median: tadalafil = 17; placebo = 16; normative score = 16) compared to healthy age-matched (65–69 years) using the same PAL test version as us [42]. As noted, there was a large and unexplained group difference in baseline PAL total errors adjusted score in our population. Some participants, especially in the placebo group at baseline, exhibited a high number of failures and hence total errors on the PAL test, indicating deficits in visual learning and episodic memory. However, their scores were much improved at the 3-month follow-up visit, hence any initial impairment was transient and improved on retest. When comparing our results on SWM to healthy elderly individuals in two other studies, there were no differences in SWM between errors score (median: tadalafil = 46; placebo = 33; healthy = 47 scores) or SWM strategy score (median: tadalafil = 37; placebo = 36; healthy = 36 scores) implying intact spatial working memory in our population [42,43]. Overall, the results from our population indicate prolonged processing speed and impairments in visual learning and memory in some individuals.

This trial has several strengths, with evenly distributed samples matched for age, sex, and educational level between groups, making them comparable. We chose a follow-up time of 3 months from baseline, which allowed us to minimize any practice effects on the different cognitive tests, although performance did improve on some. A limitation of the study was that some participants discontinued the study medication prior to study closure due to side effects, reducing the power of the per-protocol analysis. The non-compliant participants who discontinued tadalafil were on average older and more often female compared with the compliant participants, although they did not differ substantially in other baseline characteristics, CSVD severity, or cognition (Table S4 in Supplementary material). This suggests that non-compliance was unlikely to have introduced major bias in the observed cognitive outcomes. Another limitation was that we observed baseline cognitive differences between groups, with most cognitive outcomes showing slightly better scores in the placebo group (Table S5 in Supplementary material). While only a few of these differences were statistically significant, the overall directionality suggests some imbalance at baseline despite randomization into the two groups (Figure S1 in Supplementary material). The observed correlations were primarily between outcomes derived from the same cognitive tests (intra-test correlations) (Figures S2 and S3 in Supplementary material), indicating that these measures captured related cognitive domains. Given the modest sample size, the baseline imbalances most likely reflect random variation within these correlated domains rather than systematic bias. Nevertheless, such differences may have influenced subsequent change estimates and should be considered when interpreting the longitudinal findings. Further, we did a comprehensive cognitive test battery that took around 1.5 h to complete. The many tests provided a large dataset that could determine the possible benefits of tadalafil on specific cognitive domains. However, there was not very strong evidence for major cognitive deficits in the CSVD group, which would have limited the ability of tadalafil to induce improvements. Lastly, a large proportion of participants had higher levels of education, which may have caused a ceiling effect on the outcomes, making it more difficult to show improvements in cognition with tadalafil.

## 5 Conclusion and perspectives

In this ETLAS-2 sub-study, we evaluated the potential cognitive effect of daily tadalafil (20 mg) for 3 months versus placebo in participants with CSVD. We found that the total population exhibited impairments in processing speed and some deficits in visual learning and memory. Additionally, participants receiving tadalafil had a mean reaction time of 28.79 ± 13.82 milliseconds slower on the CANTAB RTI (mean simple reaction time) test compared to those receiving placebo at follow-up. This was the only outcome demonstrating a reduced reaction time with tadalafil; therefore, it could likely be a false positive. Three months of tadalafil treatment may be too short to improve cognition, as we found no effect of tadalafil on any other cognitive test. In the ETLAS-2 main study, we found a trend towards a reduction in WMH volume following tadalafil [21], and a recent longitudinal study indicates positive benefits of tadalafil on reduced mortality, cardiovascular disease, and risk of dementia [44], which warrants further exploration of tadalafil in CSVD. Therefore, we propose a more extensive study with different tadalafil dosing with at least a 1-year follow-up on clinical outcomes, including a condensed cognitive testing battery.

## Funding

The study was funded by the Novo Nordisk Foundation – Investigator Initiated Clinical Trials (grant number NNF20OC0063912), the Frimodt-Heineke Foundation (no grant number), the Foundation for Research in Neurology (no grant number), and the Herlev and Gentofte Hospital Research Fund (no grant number).

## CRediT authorship contribution statement

**Joakim Ölmestig:** Writing – review & editing, Writing – original draft, Project administration, Methodology, Investigation, Funding acquisition, Formal analysis, Data curation. **Kristian N. Mortensen:** Writing – review & editing, Formal analysis, Data curation. **Marie B. Thomas:** Writing – review & editing, Methodology, Investigation. **Birgitte Fagerlund:** Writing – review & editing, Supervision, Methodology, Investigation. **Trevor W. Robbins:** Writing – review & editing, Methodology. **Nadia Naveed:** Writing – review & editing, Investigation. **Mette M. Nordling:** Writing – review & editing, Investigation. **Hanne Christensen:** Writing – review & editing, Methodology, Investigation. **Helle K. Iversen:** Writing – review & editing, Methodology, Investigation. **Mai B. Poulsen:** Writing – review & editing, Methodology, Investigation. **Hartwig R. Siebner:** Writing – review & editing, Visualization, Methodology, Investigation. **Christina Kruuse:** Writing – review & editing, Visualization, Supervision, Project administration, Methodology, Investigation, Funding acquisition, Conceptualization.

## Declaration of competing interest

The authors declare the following financial interests/personal relationships which may be considered as potential competing interests:*Christina Kruuse was a local principal investigator in the PACIFIC (Bayer A/S), OCEANIC (Bayer A/S), and BMS A/S studies. She has received honoraria paid to the department as a speaker from Lundbeck A/S, research grants from the Novo Nordisk Foundation for the study (grant number NNF20OC0063912), and her own salary (grant number NNF18OC0031840). Hartwig R. Siebner has received honoraria as a speaker and consultant from Lundbeck A/S, Denmark, and served as editor for Neuroimage Clinical, published by Elsevier Publishers, Amsterdam, The Netherlands. He has also received royalties as a book editor from Springer Publishers, Stuttgart, Germany, Oxford University Press, Oxford, UK, and Gyldendal Publishers, Copenhagen, Denmark. He has received funding as principal investigator for the project “Precision Brain-Circuit Therapy - Precision-BCT” from Innovation Funds Denmark (grant number 9068-00025B) and the project “ADAptive and Precise Targeting of cortex-basal ganglia circuits in Parkinson´s Disease - ADAPT-PD” from Lundbeckfonden (collaborative project grant, grant number R336-2020-1035). Hanne Christensen is a member of the steering committees for the PACIFIC (Bayer A/S), OCEANIC (Bayer A/S), and ANNEXA-i (AstraZeneca A/S) trials, as well as the DSMB for Atricure. Helle K. Iversen is a local principal investigator in clinical trials conducted by Bayer A/S, BMS A/S, Janssen Research & Development, LLC, Acticor Biotech, and Biogen Idec Research Limited. Trevor W. Robbins has performed consultancy for Cambridge Cognition. Joakim Ölmestig has been a sub-investigator in clinical trials PACIFIC (Bayer A/S) and BMS A/S. The remaining authors report no conflicts.*

## Appendix Supplementary materials

Image, application 1

Image, application 2
