# Supplementary material for: Cognitive outcomes after tadalafil treatment in patients with cerebral small vessel disease: ETLAS-2 sub-study
Source: Cereb Circ Cogn Behav. 2025 Nov 11;9:100520. doi: 10.1016/j.cccb.2025.100520 (PMC12666843; doi:10.1016/j.cccb.2025.100520)
Supplement: Supplementary file 2 [file mmc2.docx]

**Supplementary material:**

Table S1: Mixed-effects models: treatment, WMH volume change, and time interactions on cognitive outcomes

| Outcome | Treatment (Estimate ± SE, p) | WMH change (Estimate ± SE, p) | Time × Treatment (Estimate ± SE, p) | Treatment × WMH change (Estimate ± SE, p) | Time × Treatment × WMH change (Estimate ± SE, p) |
| --- | --- | --- | --- | --- | --- |
| MoCA (n) | **−2.19 ± 0.80, 0.008** | −0.17 ± 0.31, 0.581 | 0.39 ± 0.62, 0.527 | −0.15 ± 0.44, 0.741 | 0.08 ± 0.33, 0.823 |
| SDMT (n) | −4.82 ± 2.63, 0.072 | −0.05 ± 1.02, 0.963 | 0.14 ± 1.25, 0.909 | −1.47 ± 1.46, 0.318 | −0.39 ± 0.67, 0.565 |
| WAIS, Digit span forward (n) | −0.60 ± 0.47, 0.207 | −0.12 ± 0.18, 0.511 | 0.13 ± 0.45, 0.781 | −0.06 ± 0.26, 0.804 | −0.10 ± 0.24, 0.677 |
| SWM, total errors (n) | **13.27 ± 6.06, 0.032** | 3.53 ± 2.33, 0.135 | -0.15 ± 4.46, 0.973 | −1.87 ± 3.36, 0.580 | 2.23 ± 2.38, 0.354 |
| PAL, total errors adjusted (n) | 8.43 ± 13.78, 0.543 | 0.49 ± 5.30, 0.927 | 9.41 ± 11.61, 0.421 | 11.97 ± 7.63, 0.121 | -0.11 ± 6.16, 0.985 |
| RTI, mean simple reaction time (ms) | 29.48 ± 23.09, 0.206 | −7.25 ± 9.36, 0.441 | 18.47 ± 21.12, 0.386 | **31.45 ± 12.99, 0.018** | **−29.19 ± 11.42, 0.014** |
| OTS, mean choices to correct (n) | 0.12 ± 0.13, 0.346 | −0.01 ± 0.05, 0.905 | 0.04 ± 0.12, 0.729 | 0.06 ± 0.07, 0.382 | −0.03 ± 0.06, 0.677 |
| RVP, A' (0-1) | −0.02 ± 0.02, 0.276 | −0.01 ± 0.01, 0.131 | 0.01 ± 0.01, 0.563 | 0.01 ± 0.01, 0.419 | −0.01 ± 0.01, 0.071 |
| Trail-making test A (s) | **9.00 ± 3.79, 0.020** | 2.06 ± 1.46, 0.162 | −1.62 ± 2.97, 0.588 | −0.59 ± 2.10, 0.781 | −1.11 ± 1.59, 0.487 |
| Trail-making test B (s) | 13.84 ± 22.76, 0.546 | −3.38 ± 8.79, 0.702 | −15.04 ± 14.05, 0.289 | 8.78 ± 12.64, 0.490 | 2.40 ± 7.51, 0.751 |
| Trail-making test total (s) | 22.84 ± 24.93, 0.363 | −1.32 ± 9.63, 0.891 | −16.66 ± 14.01, 0.240 | 8.19 ± 13.86, 0.557 | 1.29 ± 7.49, 0.864 |
| Trail-making test B/A ratio | −0.13 ± 0.34, 0.702 | −0.16 ± 0.13, 0.228 | −0.14 ± 0.34, 0.688 | 0.22 ± 0.19, 0.254 | 0.14 ± 0.18, 0.456 |
| Verbal fluency, F (n) | −2.22 ± 1.23, 0.075 | 0.06 ± 0.47, 0.888 | 1.17 ± 1.23, 0.345 | −0.35 ± 0.68, 0.612 | 0.09 ± 0.66, 0.897 |
| Verbal fluency, A (n) | **−2.13 ± 0.93, 0.025** | −0.14 ± 0.36, 0.689 | 0.71 ± 0.95, 0.459 | −0.07 ± 0.51, 0.889 | 0.43 ± 0.51, 0.399 |
| Verbal fluency, S (n) | −1.45 ± 1.32, 0.276 | −0.06 ± 0.51, 0.900 | 0.33 ± 1.14, 0.773 | −0.23 ± 0.73, 0.758 | 1.00 ± 0.61, 0.106 |
| Verbal fluency, animal (n) | **−3.81 ± 1.61, 0.021** | −0.08 ± 0.62, 0.899 | 1.70 ± 1.40, 0.231 | −0.97 ± 0.89, 0.282 | 0.55 ± 0.75, 0.470 |
| Verbal fluency, total (n) | **−9.61 ± 3.93, 0.018** | −0.22 ± 1.52, 0.885 | 3.91 ± 2.34, 0.101 | −1.61 ± 2.19, 0.464 | 2.06 ± 1.25, 0.105 |
| WAIS IV, Digit span backwards (n) | −0.52 ± 0.56, 0.353 | 0.02 ± 0.21, 0.915 | 0.21 ± 0.54, 0.695 | −0.16 ± 0.31, 0.602 | −0.41 ± 0.29, 0.160 |
| WAIS IV, Digit span ordering (n) | −0.48 ± 0.62, 0.444 | 0.11 ± 0.24, 0.648 | −0.31 ± 0.52, 0.548 | −0.34 ± 0.34, 0.323 | −0.26 ± 0.28, 0.361 |
| WAIS IV, Letter number sequence (n) | **−3.28 ± 1.13, 0.005** | 0.08 ± 0.44, 0.864 | **2.48 ± 0.89, 0.007** | −0.93 ± 0.63, 0.145 | −0.08 ± 0.47, 0.871 |
| WAIS IV, total score (n) | **−4.88 ± 2.26, 0.035** | 0.10 ± 0.87, 0.907 | 2.18 ± 1.39, 0.123 | −1.51 ± 1.26, 0.233 | −0.87 ± 0.74, 0.249 |
| SWM, between errors (n) | **4.30 ± 2.03, 0.038** | −0.21 ± 0.78, 0.790 | −0.71 ± 1.89, 0.709 | 0.24 ± 1.12, 0.829 | −0.53 ± 1.01, 0.604 |
| SWM, strategy (8-56) | **12.73 ± 5.98, 0.037** | 3.03 ± 2.30, 0.193 | 0.58 ± 4.38, 0.894 | −1.43 ± 3.32, 0.667 | 1.90 ± 2.34, 0.420 |
| PAL, total trials (n) | 2.75 ± 2.91, 0.350 | 0.28 ± 1.12, 0.804 | 1.69 ± 2.01, 0.410 | 1.88 ± 1.62, 0.249 | 0.30 ± 1.07, 0.778 |
| PAL, first trial memory score (n) | −1.52 ± 1.36, 0.270 | −0.40 ± 0.52, 0.453 | −0.85 ± 1.10, 0.441 | −0.60 ± 0.76, 0.426 | −0.39 ± 0.58, 0.512 |
| RTI, mean simple movement time (ms) | −3.67 ± 32.67, 0.911 | 30.25 ± 13.23, 0.025 | −12.88 ± 20.87, 0.540 | −36.57 ± 18.38, 0.050 | 30.51 ± 16.02, 0.062 |
| RTI, mean five choice reaction time (ms) | 26.64 ± 24.75, 0.285 | 7.12 ± 10.06, 0.481 | 17.71 ± 16.95, 0.301 | 6.53 ± 13.93, 0.641 | 4.38 ± 13.02, 0.738 |
| RTI, mean five choice movement time (ms) | 56.10 ± 33.97, 0.102 | 23.72 ± 13.89, 0.091 | 15.15 ± 25.77, 0.559 | −16.70 ± 19.13, 0.385 | 8.06 ± 19.81, 0.686 |
| OTS, problems solved on first choice (n) | **−2.11 ± 0.92, 0.024** | −0.05 ± 0.35, 0.894 | 1.01 ± 0.54, 0.069 | −0.52 ± 0.509, 0.312 | 0.21 ± 0.424, 0.618 |
| OTS, mean latency to correct (ms) | −1478.98 ± 6223.40, 0.813 | 1323.22 ± 2388.05, 0.581 | −4304.93 ± 3924.23, 0.278 | −1773.52 ± 3442.73, 0.608 | 3657.94 ± 3084.05, 0.241 |
| RVP, mean latency (ms) | 16.73 ± 38.48, 0.665 | 32.79 ± 20.24, 0.109 | −5.71 ± 29.88, 0.849 | −40.17 ± 25.09, 0.113 | 48.87 ± 27.72, 0.084 |

Results in **bold** are statistically significant with a p-value < 0.05. All models include age and sex as covariates. Mixed-effects models are done as post-hoc analyses.

Table S2: Mixed-effects models: treatment, CBF change, and time interactions on cognitive outcomes

| Outcome | Treatment (Estimate ± SE, p) | CBF change (Estimate ± SE, p) | Time × Treatment (Estimate ± SE, p) | Treatment × CBF change (Estimate ± SE, p) | Time × Treatment × CBF change (Estimate ± SE, p) |
| --- | --- | --- | --- | --- | --- |
| MoCA (n) | -1.46 ± 0.96, 0.135 | 0.09 ± 0.20, 0.649 | 0.49 ± 0.82, 0.555 | -0.14 ± 0.26, 0.580 | -0.03 ± 0.22, 0.886 |
| SDMT (n) | -5.17 ± 3.44, 0.141 | 0.49 ± 0.73, 0.503 | 0.29 ± 1.71, 0.867 | -1.03 ± 0.93, 0.272 | 0.13 ± 0.46, 0.776 |
| WAIS, Digit span forward (n) | -0.80 ± 0.53, 0.141 | 0.02 ± 0.11, 0.837 | -0.18 ± 0.59, 0.759 | -0.05 ± 0.14, 0.711 | 0.15 ± 0.16, 0.344 |
| SWM, total errors (n) | **13.86 ± 7.62, 0.076** | -1.52 ± 1.61, 0.350 | 3.43 ± 5.58, 0.543 | 2.43 ± 2.05, 0.244 | 0.05 ± 1.52, 0.975 |
| PAL, total errors adjusted (n) | 0.86 ± 16.74, 0.959 | -0.21 ± 3.54, 0.953 | 0.24 ± 14.37, 0.987 | 1.13 ± 4.51, 0.803 | 2.69 ± 3.88, 0.493 |
| RTI, mean simple reaction time (ms) | 3.51 ± 27.66, 0.900 | -7.64 ± 5.85, 0.197 | 21.15 ± 24.64, 0.396 | **24.74 ± 7.46, 0.002** | **-14.14 ± 6.65, 0.040** |
| OTS, mean choices to correct (n) | 0.21 ± 0.17, 0.242 | -0.01 ± 0.04, 0.778 | -0.02 ± 0.12, 0.900 | -0.02 ± 0.05, 0.729 | **0.07 ± 0.03, 0.039** |
| RVP, A' (0-1) | **-0.04 ± 0.02, 0.046** | 0.00 ± 0.00, 0.272 | 0.01 ± 0.01, 0.647 | -0.01 ± 0.01, 0.264 | -0.01 ± 0.00, 0.061 |
| Trail-making test A (s) | **10.41 ± 4.61, 0.029** | -1.49 ± 0.98, 0.134 | -2.73 ± 3.31, 0.415 | **2.53 ± 1.24, 0.047** | -1.55 ± 0.90, 0.094 |
| Trail-making test B (s) | 14.85 ± 31.33, 0.638 | -3.37 ± 6.64, 0.615 | -4.80 ± 18.22, 0.794 | 5.32 ± 8.44, 0.532 | 8.29 ± 4.96, 0.103 |
| Trail-making test total (s) | 25.26 ± 33.85, 0.460 | -4.86 ± 7.17, 0.502 | -7.53 ± 18.14, 0.680 | 7.85 ± 9.12, 0.395 | 6.75 ± 4.94, 0.180 |
| Trail-making test B/A ratio | -0.24 ± 0.48, 0.610 | 0.06 ± 0.10, 0.573 | 0.20 ± 0.43, 0.637 | -0.08 ± 0.13, 0.528 | **0.29 ± 0.12, 0.018** |
| Verbal fluency, F (n) | -2.71 ± 1.62, 0.100 | 0.41 ± 0.34, 0.233 | 1.66 ± 1.60, 0.308 | -0.47 ± 0.44, 0.289 | -0.18 ± 0.44, 0.687 |
| Verbal fluency, A (n) | **-2.48 ± 1.16, 0.037** | 0.08 ± 0.25, 0.731 | 0.91 ± 1.27, 0.481 | 0.07 ± 0.31, 0.821 | -0.57 ± 0.35, 0.109 |
| Verbal fluency, S (n) | -0.97 ± 1.49, 0.516 | 0.42 ± 0.32, 0.191 | -0.31 ± 1.28, 0.808 | -0.39 ± 0.40, 0.342 | -0.06 ± 0.35, 0.867 |
| Verbal fluency, animal (n) | -3.59 ± 2.17, 0.105 | 0.37 ± 0.46, 0.429 | 1.94 ± 1.63, 0.241 | -0.53 ± 0.58, 0.369 | -0.22 ± 0.44, 0.622 |
| Verbal fluency, total (n) | -9.75 ± 4.97, 0.056 | 1.28 ± 1.05, 0.230 | 4.19 ± 2.96, 0.166 | -1.31 ± 1.34, 0.332 | -1.03 ± 0.81, 0.211 |
| WAIS IV, Digit span backwards (n) | -0.76 ± 0.64, 0.238 | 0.17 ± 0.14, 0.211 | 0.14 ± 0.67, 0.838 | -0.10 ± 0.17, 0.580 | -0.29 ± 0.18, 0.122 |
| WAIS IV, Digit span ordering (n) | -1.24 ± 0.74, 0.102 | 0.16 ± 0.16, 0.318 | 0.02 ± 0.63, 0.975 | -0.24 ± 0.20, 0.233 | -0.05 ± 0.17, 0.763 |
| WAIS IV, Letter number sequence (n) | **-2.85 ± 1.40, 0.047** | 0.33 ± 0.30, 0.272 | 1.53 ± 1.02, 0.143 | -0.45 ± 0.38, 0.236 | 0.09 ± 0.28, 0.738 |
| WAIS IV, total score (n) | **-5.70 ± 2.71, 0.042** | 0.70 ± 0.57, 0.233 | 1.13 ± 1.73, 0.518 | -0.85 ± 0.73, 0.251 | -0.10 ± 0.47, 0.833 |
| SWM, between errors (n) | 13.46 ± 7.54, 0.081 | -1.64 ± 1.60, 0.310 | 3.90 ± 5.42, 0.476 | 2.59 ± 2.03, 0.209 | -0.20 ± 1.47, 0.894 |
| SWM, strategy (8-56) | 5.08 ± 2.54, 0.051 | -0.73 ± 0.54, 0.182 | 0.99 ± 2.36, 0.679 | 1.03 ± 0.69, 0.139 | 0.28 ± 0.64, 0.668 |
| PAL, total trials (n) | 0.90 ± 2.92, 0.759 | 0.02 ± 0.62, 0.979 | 0.13 ± 2.22, 0.954 | 0.13 ± 0.79, 0.871 | 0.34 ± 0.60, 0.574 |
| PAL, first trial memory score (n) | -0.06 ± 1.72, 0.974 | -0.09 ± 0.36, 0.805 | -0.08 ± 1.42, 0.956 | -0.06 ± 0.46, 0.902 | -0.35 ± 0.38, 0.368 |
| RTI, mean simple movement time (ms) | -21.29 ± 32.38, 0.513 | -11.72 ± 6.85, 0.093 | -9.26 ± 32.39, 0.777 | **29.49 ± 8.74, 0.001** | -10.53 ± 8.75, 0.237 |
| RTI, mean five choice reaction time (ms) | 17.43 ± 26.46, 0.514 | -0.56 ± 5.60, 0.921 | -6.76 ± 18.97, 0.724 | 10.14 ± 7.13, 0.162 | **10.56 ± 5.12, 0.046** |
| RTI, mean five choice movement time (ms) | 72.73 ± 37.40, 0.057 | -15.75 ± 7.90, 0.051 | **-97.97 ± 40.47, 0.021** | 16.35 ± 10.09, 0.111 | 6.40 ± 10.94, 0.562 |
| OTS, problems solved on first choice (n) | -1.77 ± 0.99, 0.081 | -0.14 ± 0.21, 0.508 | 0.95 ± 0.89, 0.289 | 0.44 ± 0.27, 0.103 | **-0.77 ± 0.24, 0.003** |
| OTS, mean latency to correct (ms) | -1154 ± 7696, 0.881 | 1731 ± 1628, 0.293 | 2373 ± 6834, 0.731 | -1323 ± 2075, 0.527 | -1626 ± 1846, 0.384 |
| RVP, mean latency (ms) | **91.90 ± 38.13, 0.020** | -14.32 ± 8.04, 0.081 | **-74.12 ± 36.31, 0.049** | 10.69 ± 10.21, 0.300 | 3.41 ± 9.86, 0.732 |

Results in **bold** are statistically significant with a p-value < 0.05. All models include age and sex as covariates. Mixed-effects models are done as post-hoc analyses.

Table S3: Reaction times in tadalafil users

| Group | Baseline RTI | Follow-up RTI | Change (Δ) |
| --- | --- | --- | --- |
| Per-protocol tadalafil | 376 (337, 400) (n=28) | 392 (337, 427) (n=27) | 7.67 (-32.11, 46.38) (n=27) |
| Non-compliant / dropouts | 380 (349, 395) (n=10) | 376 (371, 489) (n=5) | -0.78 (-0.89, 49.22) (n=5) |

Reaction time is from CANTAB - RTI, mean simple reaction time. Values are presented as median (Q1, Q3). These results are done as post-hoc analyses.

Table S4: Baseline characteristics of tadalafil-treated participants who completed the study versus those who discontinued tadalafil

| Characteristic | Discontinued tadalafil  N = 10^1^ | Completed tadalafil  N = 28^1^ | p-value^2^ |
| --- | --- | --- | --- |
| **Background data** |  |  |  |
| Age | 76 (73, 80) | 68 (59, 72) | 0.005 |
| Sex |  |  | 0.062 |
| Female | 6 (60%) | 7 (25%) |  |
| Male | 4 (40%) | 21 (75%) |  |
| Highest educational level |  |  | 0.7 |
| Primary school | 1 (10%) | 2 (7.1%) |  |
| Secondary school | 0 (0%) | 1 (3.6%) |  |
| Vocational education | 1 (10%) | 5 (18%) |  |
| Short higher education (2-3.5 years) | 1 (10%) | 3 (11%) |  |
| Intermediate higher education (3.5-4 years) | 6 (60%) | 9 (32%) |  |
| Long higher education (5-6 years) | 1 (10%) | 8 (29%) |  |
| Hypertension | 6 (60%) | 22 (79%) | 0.4 |
| Type 2 diabetes | 0 (0%) | 3 (11%) | 0.6 |
| Hypercholesterolemia | 10 (100%) | 27 (96%) | >0.9 |
| Smoking status |  |  | 0.088 |
| Never | 7 (70%) | 8 (29%) |  |
| Current | 0 (0%) | 5 (18%) |  |
| Previous | 3 (30%) | 15 (54%) |  |
| Ischemic stroke | 4 (40%) | 20 (71%) | 0.13 |
| Transient ischemic attack | 6 (60%) | 8 (29%) | 0.13 |
| Periventricular Fazekas scale |  |  | 0.9 |
| 0 | 0 (0%) | 2 (7.1%) |  |
| 1 | 3 (30%) | 9 (32%) |  |
| 2 | 4 (40%) | 12 (43%) |  |
| 3 | 3 (30%) | 5 (18%) |  |
| Deep Fazekas scale |  |  | 0.7 |
| 0 | 1 (10%) | 2 (7.1%) |  |
| 1 | 2 (20%) | 10 (36%) |  |
| 2 | 4 (40%) | 11 (39%) |  |
| 3 | 3 (30%) | 5 (18%) |  |
| **Mental well-being and premorbid intelligence** |  |  |  |
| BDI-II | 5.5 (3.0, 6.5) (n=8) | 5.0 (2.0, 10.0) (n=27) | >0.9 |
| FSS | 3.06 (2.44, 4.61) (n=8) | 3.44 (2.33, 5.78) (n=27) | 0.8 |
| WHO-5 | 72 (64, 78) (n=8) | 72 (52, 84) (n=27) | >0.9 |
| IQCODE | 3.25 (3.09, 3.39) (n=8) | 3.06 (3.00, 3.42) (n=27) | 0.3 |
| DART | 29 (21, 30) | 22 (13, 28) (n=27) | 0.14 |
| **Primary cognitive outcomes** |  |  |  |
| MoCA (0–30) | 27.00 (27.00, 29.00) | 26.00 (23.50, 27.00) | 0.032 |
| SDMT (0–110) | 37 (33, 38) | 35 (31, 44) | 0.9 |
| WAIS IV, Digit span forward (0–16) | 7.50 (7.00, 9.00) | 7.50 (7.00, 8.00) | >0.9 |
| SWM, total errors (n) | 53 (39, 61) | 47 (20, 59) | 0.6 |
| PAL, total errors adjusted (n) | 72 (29, 123) | 33 (14, 78) | 0.2 |
| RTI, mean simple reaction time (ms) | 380 (340, 400) | 376 (337, 400) | 0.9 |
| OTS, mean choices to correct (n) | 1.87 (1.60, 2.33) (n=9) | 1.40 (1.27, 1.87) | 0.082 |
| RVP, A' (n, 0–1) | 0.84 (0.79, 0.87) (n=9) | 0.87 (0.85, 0.91) (n=26) | 0.070 |
| **Secondary cognitive outcomes** |  |  |  |
| Trail-making test A (s) | 41 (33, 53) | 39 (33, 54) | 0.9 |
| Trail-making test B (s) | 106 (85, 148) | 110 (80, 145) | >0.9 |
| Trail-making test total (s) | 147 (124, 198) | 153 (112, 199) | >0.9 |
| Trail-making test B/A ratio | 2.52 (2.18, 3.76) | 2.76 (2.37, 3.38) | 0.8 |
| Verbal fluency, F (n) | 10.0 (8.0, 15.0) | 11.0 (8.5, 12.0) | 0.8 |
| Verbal fluency, A (n) | 9.00 (7.00, 11.00) | 7.00 (6.00, 9.00) | 0.13 |
| Verbal fluency, S (n) | 12.5 (8.0, 15.0) | 11.0 (9.0, 13.0) | 0.4 |
| Verbal fluency, animals (n) | 15.0 (12.0, 19.0) | 17.5 (14.5, 20.5) | 0.3 |
| Verbal fluency, total (n) | 49 (35, 55) | 49 (40, 52) | 0.8 |
| WAIS IV, Digit span backwards (n, 0–16) | 6.50 (5.00, 8.00) | 6.00 (6.00, 7.50) | 0.8 |
| WAIS IV, Digit span ordering (n, 0–16) | 7.00 (6.00, 8.00) | 6.00 (5.00, 8.00) | 0.14 |
| WAIS IV, Letter number sequence (n, 0–30) | 16.0 (15.0, 18.0) | 16.0 (13.0, 17.0) | 0.3 |
| WAIS IV, total score (n, 0–78) | 38 (35, 41) | 35 (32, 41) | 0.3 |
| SWM, between errors (n) | 51 (33, 58) | 46 (18, 59) | 0.6 |
| SWM, strategy (8-56) | 36.5 (36.0, 38.0) | 37.0 (32.5, 41.0) | 0.6 |
| PAL, total trials (n) | 24 (16, 28) | 18 (13, 23) | 0.2 |
| PAL, first trial memory score (n) | 11.5 (10.0, 16.0) | 16.5 (13.0, 19.5) | 0.062 |
| RTI, mean simple movement time (ms) | 482 (361, 500) | 471 (388, 574) | 0.5 |
| RTI, mean five choice reaction time (ms) | 447 (426, 519) | 399 (367, 430) | 0.076 |
| RTI, mean five choice movement time (ms) | 487 (403, 527) | 487 (444, 569) | 0.6 |
| OTS, problems solved on first choice (n) | 8.5 (5.0, 9.0) | 8.0 (7.0, 11.5) | 0.4 |
| OTS, mean latency to correct (ms) | 39,214 (37,247, 50,435) (n=9) | 38,722 (25,118, 54,531) | 0.7 |
| RVP, mean latency | 605 (541, 762) (n=9) | 547 (441, 630) (n=26) | 0.2 |

^1^Median (Q1, Q3). ^2^Wilcoxon rank sum test; Fisher's exact test; Wilcoxon rank sum exact test

Table S5: Baseline cognitive outcomes by treatment groups

| Outcome | Placebo (mean) | Tadalafil (mean) | p-value | Test |
| --- | --- | --- | --- | --- |
| MoCA (0–30) | 26.87 | 25.18 | **0.0129** | Wilcoxon |
| SDMT (0–110) | 38.16 | 37.04 | 0.6950 | t-test |
| WAIS IV, Digit span forward (0–16) | 8.00 | 7.75 | 0.5540 | Wilcoxon |
| SWM, total errors (n) | 33.71 | 41.50 | 0.2090 | t-test |
| PAL, total errors adjusted (n) | 59.84 | 53.86 | 0.6540 | Wilcoxon |
| RTI, mean simple reaction time (ms) | 383.88 | 390.90 | 0.3820 | Wilcoxon |
| OTS, mean choices to correct (n) | 1.49 | 1.56 | 0.2170 | Wilcoxon |
| RVP, A' (n, 0–1) | 0.89 | 0.88 | 0.2600 | Wilcoxon |
| Trail-making test A (s) | 39.48 | 44.39 | 0.1120 | Wilcoxon |
| Trail-making test B (s) | 110.80 | 123.50 | *0.0722* | Wilcoxon |
| Trail-making test total (s) | 149.00 | 167.89 | *0.0686* | Wilcoxon |
| Trail-making test B/A ratio | 2.88 | 2.83 | 0.7150 | Wilcoxon |
| Verbal fluency, F (n) | 11.94 | 10.29 | 0.1020 | t-test |
| Verbal fluency, A (n) | 9.16 | 7.50 | *0.0584* | t-test |
| Verbal fluency, S (n) | 12.06 | 11.11 | 0.3900 | t-test |
| Verbal fluency, animals (n) | 20.32 | 17.82 | 0.1330 | t-test |
| Verbal fluency, total (n) | 53.48 | 46.71 | *0.0647* | t-test |
| WAIS IV, Digit span backwards (n, 0–16) | 6.84 | 6.50 | 0.5630 | Wilcoxon |
| WAIS IV, Digit span ordering (n, 0–16) | 6.65 | 6.50 | 0.7990 | t-test |
| WAIS IV, Letter number sequence (n, 0–30) | 16.97 | 14.50 | **0.0149** | Wilcoxon |
| WAIS IV, total score (n, 0–78) | 38.45 | 35.25 | 0.1040 | t-test |
| SWM, between errors (n) | 33.00 | 40.71 | 0.2060 | t-test |
| SWM, strategy (8-56) | 32.61 | 36.32 | **0.0427** | Wilcoxon |
| PAL, total trials (n) | 19.94 | 19.71 | 0.7840 | Wilcoxon |
| PAL, first trial memory score (n) | 15.71 | 15.75 | 0.9740 | t-test |
| RTI, mean simple movement time (ms) | 549.75 | 490.62 | 0.2680 | Wilcoxon |
| RTI, mean five choice reaction time (ms) | 419.44 | 425.64 | 0.7050 | Wilcoxon |
| RTI, mean five choice movement time (ms) | 482.48 | 500.11 | 0.5580 | t-test |
| OTS, problems solved on first choice (n) | 9.58 | 8.50 | 0.1940 | Wilcoxon |
| OTS, mean latency to correct (ms) | 46,021.45 | 42,502.24 | 0.6490 | Wilcoxon |
| RVP, mean latency | 533.15 | 543.20 | 0.4970 | Wilcoxon |

The table presents mean baseline values between the groups and test if they are different using t-test or Wilcoxon test depending on normality of the dataset. P-values in **bold** are statistically significant. P-values in *italic* are near-significant.

Figure S1: Adjusted baseline differences between groups
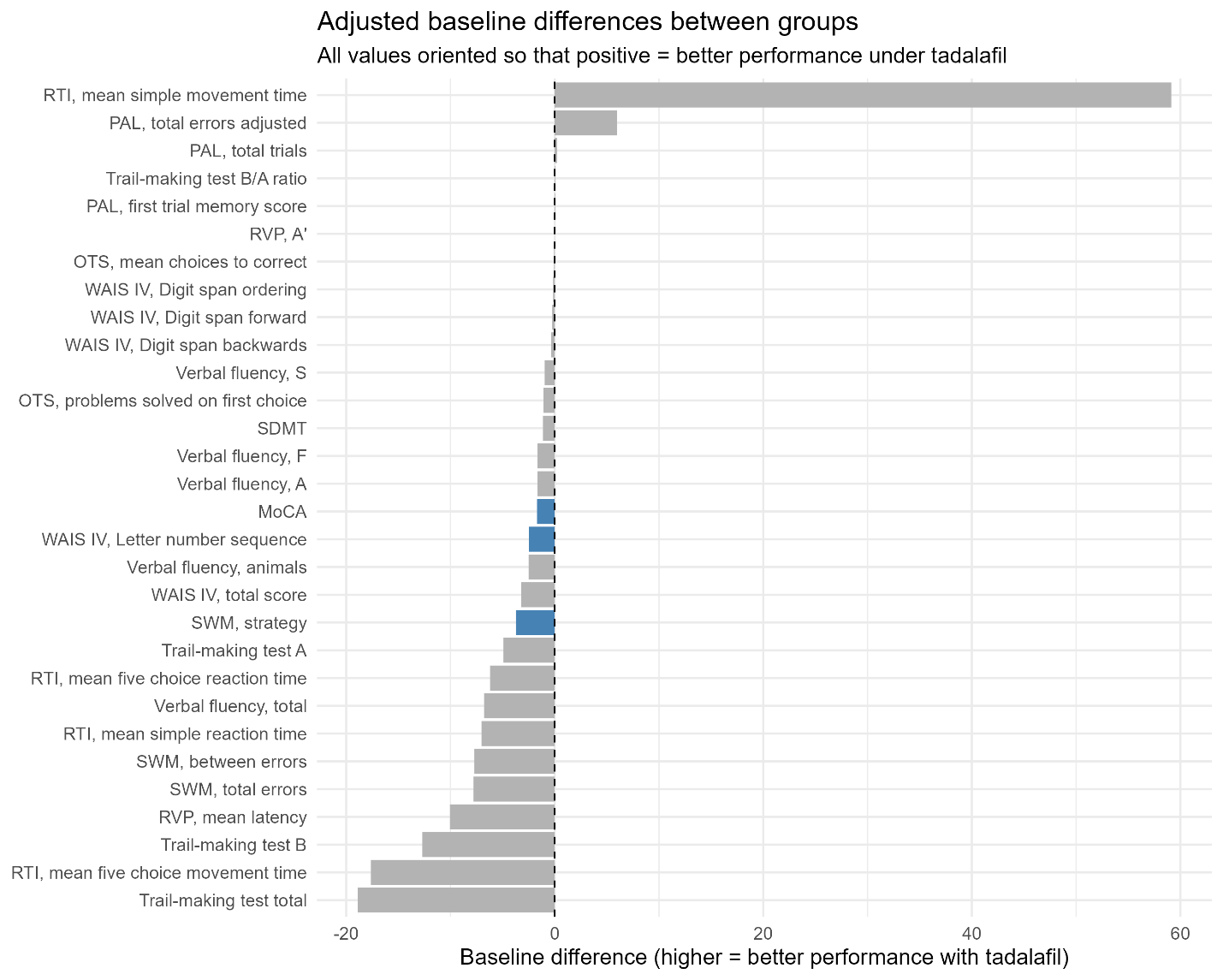


Baseline differences are calculated as the mean tadalafil baseline value minus the mean placebo baseline value. All values are oriented so that positive values equal better performance for tadalafil. Blue bars are statistically significant. The outcome ´OTS, mean latency to correct` has been omitted from the figure due to scale differences.


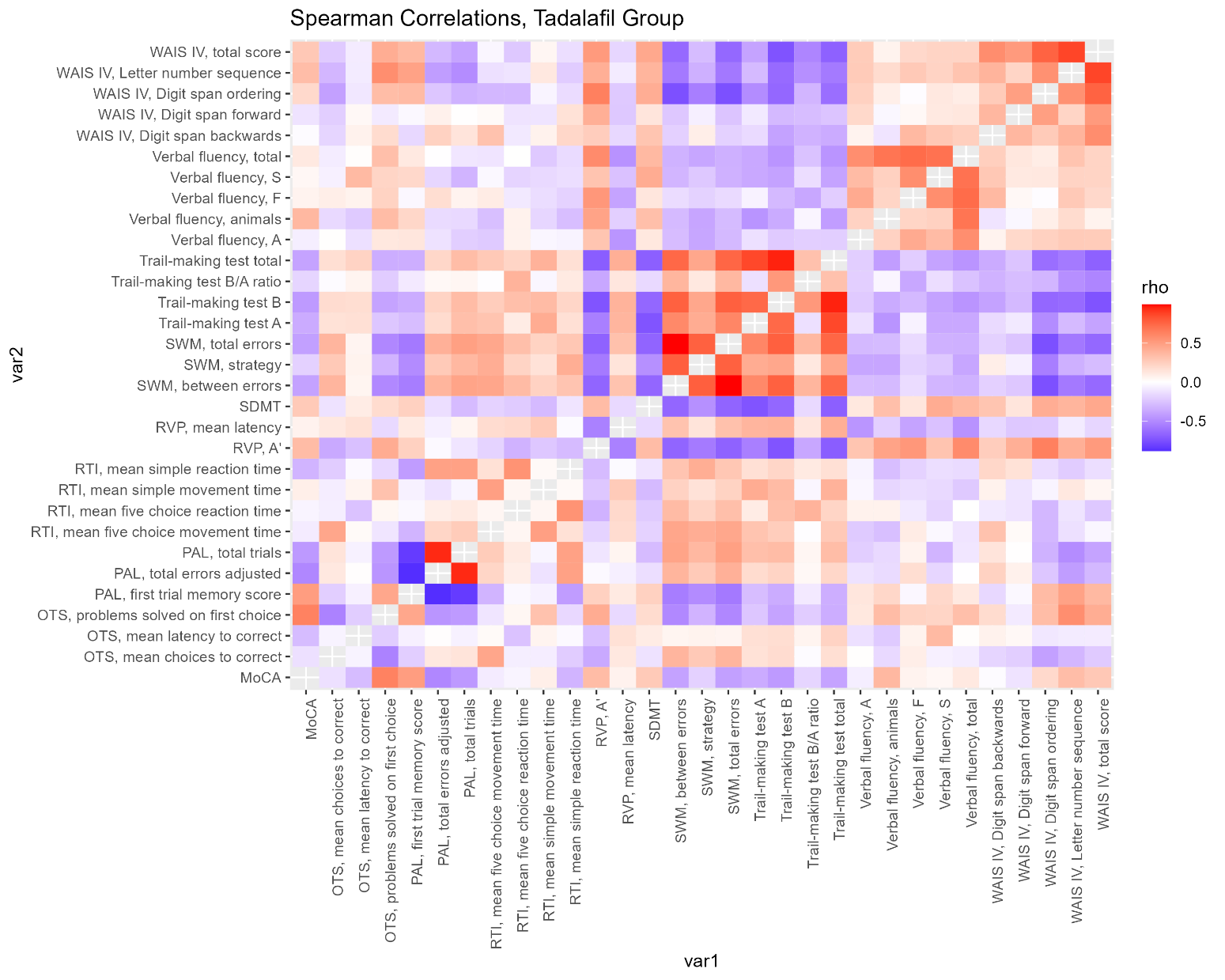
Figure S2. Spearman correlations, Tadalafil group


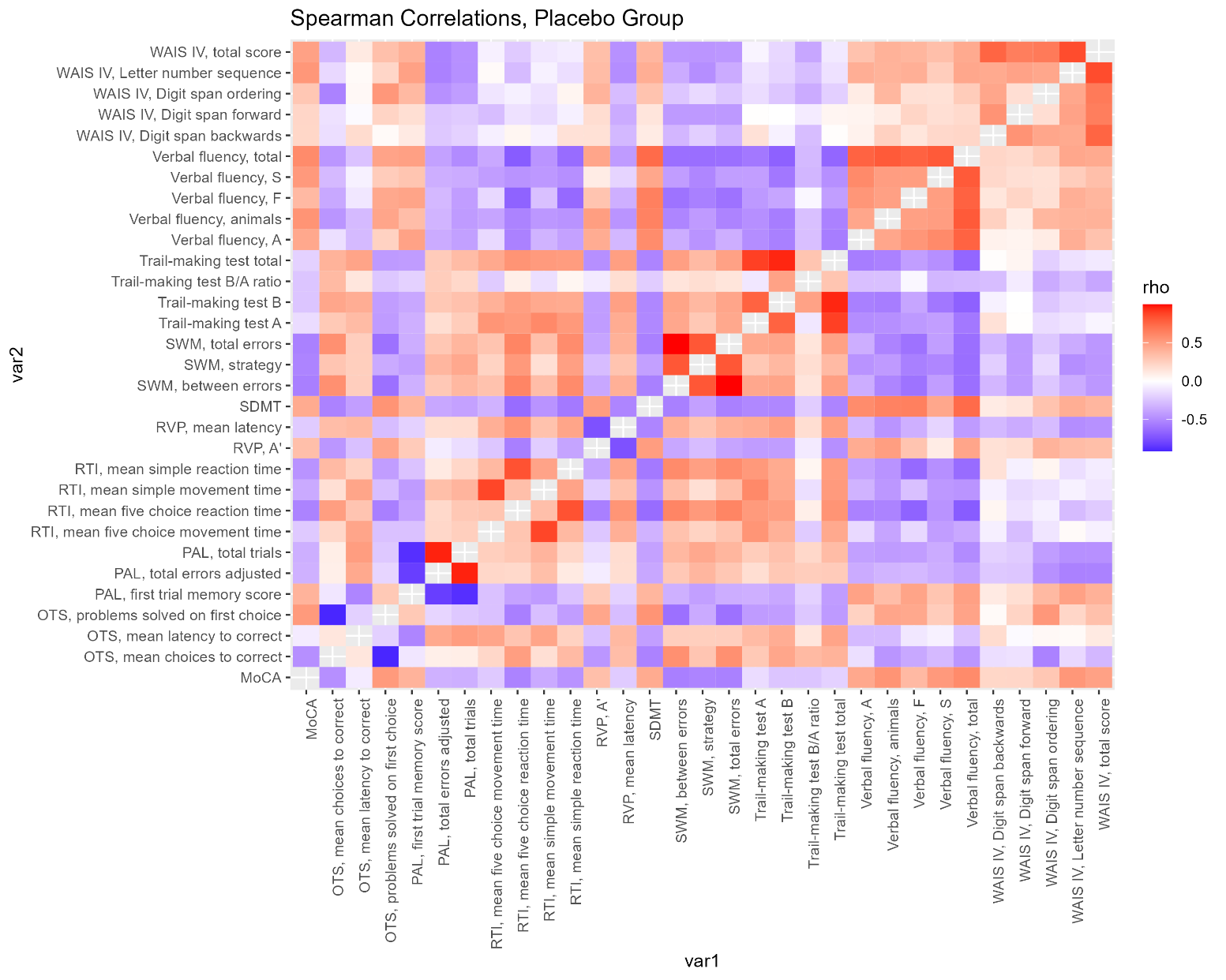
Figure S3. Spearman correlations, Placebo group
